# Impact of clinicopathologic features on leptomeningeal metastasis from lung adenocarcinoma and treatment efficacy with epidermal growth factor receptor tyrosine kinase inhibitor

**DOI:** 10.1111/1759-7714.13296

**Published:** 2020-01-07

**Authors:** Byoung Soo Kwon, Young Hyun Cho, Shin‐Kyo Yoon, Dae Ho Lee, Sang‐We Kim, Do Hoon Kwon, Jae Cheol Lee, Chang‐Min Choi

**Affiliations:** ^1^ Department of Pulmonology and Critical Care Medicine, Asan Medical Center University of Ulsan College of Medicine Seoul South Korea; ^2^ Department of Neurosurgery, Asan Medical Center University of Ulsan College of Medicine Seoul South Korea; ^3^ Department of Oncology, Asan Medical Center University of Ulsan College of Medicine Seoul South Korea

**Keywords:** Adenocarcinoma, EGFR‐TKI, intrathecal (IT), leptomeningeal metastasis, lung cancer

## Abstract

**Background:**

We investigated the risk factors for leptomeningeal carcinomatosis (LMC) and compared clinical efficacies of various treatment modalities including intrathecal (IT) chemotherapy in patients with lung adenocarcinoma harboring epidermal growth factor receptor (EGFR) mutations.

**Methods:**

Using clinical research data from the Asan Medical Center, we retrospectively analyzed data of patients diagnosed with LMC, confirmed via cerebrospinal fluid (CSF) analysis from January 2008 to December 2017.

**Results:**

We identified 1189 patients with lung adenocarcinoma harboring EGFR mutations. Among these, 9.8% had a median duration of 13.5 (interquartile range [IQR] 6.8–23.6) months from the initial lung cancer diagnosis to LMC occurrence. Younger age (hazard ratio [HR] 1.043, *P* < 0.001), initial metastatic disease (HR 3.768, *P* < 0.001), and metastasis to the brain (HR 8.682, *P* < 0.001) or lung (HR 2.317, *P* = 0.004) were risk factors associated with LMC. Median survival duration from LMC diagnosis was 3.8 (IQR 1.5–8.6) months. Eastern Cooperative Oncology Group performance status score ≤ 2 (HR 0.505, *P* = 0.007) and insertion of Ommaya reservoir (HR 0.445, *P* = 0.005) were associated with longer survival. EGFR‐tyrosine kinase inhibitor (TKI) conferred survival benefits compared to cytotoxic chemotherapy or best supportive care (HR 2.222, *P* = 0.018; HR 5.638, *P* < 0.001, respectively). Although IT chemotherapy showed no survival benefit, it was associated with improved neurologic symptoms and signs and CSF negative conversion.

**Conclusions:**

Younger age, initial diagnosis of metastatic disease, and metastasis to the brain or different lobes were associated with LMC in patients with EGFR‐mutant lung adenocarcinoma. Therapeutic interventions including EGFR‐TKIs, cytotoxic chemotherapy, or Ommaya reservoir, and good performance status were related to favorable survival outcomes.

**Key points:**

Age and disease status were associated with LMC in patients with EGFR‐mutant adenocarcinoma, and EGFR‐TKI, Ommaya reservoir, and good performance status were related to survival benefit.

## Introduction

Lung cancer is the leading cause of cancer‐related mortality worldwide, with a five‐year survival rate of less than 20%.^1^, ^2^ However, with the introduction of epidermal growth factor receptor‐tyrosine kinase inhibitor (EGFR‐TKI) treatment, lung cancer mortality has markedly improved in comparison with that of cytotoxic chemotherapy.^3^, ^4^, ^5^ In Korea, the incidence and survival outcome of lung cancer has changed over time in a manner similar to that of other countries.^6^, ^7^, ^8^

Leptomeningeal carcinomatosis (LMC) is a devastating complication of advanced lung cancer. Advances in treatment have resulted in survival prolongation among patients with lung cancer, but this has resulted in a corresponding increase in the incidence of LMC.^9^ The incidence of LMC among patients with non‐small cell lung cancer (NSCLC) has been previously reported to be 3%–5%,^10^ and LMC more frequently occurs in patients with EGFR mutations (in up to 20% of patients) than in patients with EGFR wild type.^11^ The main challenge in treating LMC is poor penetration of chemotherapeutic agents through the blood‐brain barrier. Therefore, the median survival of patients with LMC remains low, at only three–six months.^12^

To date, no standard therapeutic regimen for LMC has been established and treatment outcomes have not been evaluated in patients with lung cancer because of its rarity and heterogeneity.^9^ In particular, in cases of adenocarcinoma there are limited data on the effectiveness of EGFR‐TKI and other chemotherapeutic agents. In addition, the role is unknown of the Ommaya reservoir which is an intraventricular catheter used for intrathecal (IT) chemotherapy or for controlling increased intracranial pressure (IICP). Taking this into consideration, our study aimed to investigate the incidence and risk factors for LMC and to compare the clinical efficacies of various treatment modalities and clinical feasibility of the Ommaya reservoir, focusing on patients with lung adenocarcinoma harboring EGFR mutations.

## Methods

### Study patients

From January 2008 to December 2017, clinical data of patients with an initial diagnosis of metastatic or recurrent lung adenocarcinoma were extracted using ABLE (Asan Biomedical Research Environment), the deidentified clinical research data warehouse of Asan Medical Center, a 2700 bed tertiary referral hospital in Seoul, South Korea.^13^, ^14^ Among these patients, those with EGFR mutations were screened. Patients were selected who were clinically or radiologically suspected of having LMC and where the results of cerebrospinal fluid (CSF) analysis were available. We then included patients with confirmed CSF malignancy. We excluded the following patients: (i) those younger than 18 years; (ii) those receiving immuno‐oncology therapy; and (iii) those who had received IT chemotherapy for less than one month. To evaluate the efficacy of IT chemotherapy, only patients treated with methotrexate were included in the analysis.

The study protocol was approved by the institutional review board of Asan Medical Center (IRB No. 2017–0017), which waived the requirement for informed consent because of the retrospective nature of the analysis.

### Risk factors for LMC

We collected data regarding the exons with EGFR mutations and metastatic lesions to evaluate the risk factors for LMC. EGFR mutation was confirmed by nested polymerase chain reaction (PCR) amplification of the individual exons 18, 19, 20, and 21. Metastatic lesions were categorized as follows: brain, bone, liver, lung, pleura, and adrenal gland.

### Treatment modalities and outcome measures

The treatment modalities consisted of EGFR‐TKIs, cytotoxic chemotherapy, IT chemotherapy, and best supportive care. We further subdivided patients who received EGFR‐TKIs to those treated with first‐generation (gefitinib, erlotinib) and third‐generation (osimertinib) EGFR‐TKIs. IT chemotherapy was administered in combination with other anticancer treatments or independently.

Overall survival (OS) was defined as duration from the time of LMC diagnosis to any cause of death. Neurologic outcome measures included two categories: (i) symptom improvement and (ii) cytologic conversion. Neurologic symptoms included headache, signs of cauda equina, dizziness, seizure, altered mentality, and memory impairment. We evaluated the proportion of patients with improvement of neurologic symptoms, according to those with an Ommaya reservoir and those treated with IT chemotherapy. Negative conversion of CSF cytology was defined as no evidence of malignant cells or atypical cells in a follow‐up CSF study after treatment initiation.

### Statistical analysis

The primary endpoint was OS for each treatment modality, including EGFR‐TKIs, cytotoxic chemotherapy, and best supportive care. Secondary endpoints were neurologic outcomes of IT chemotherapy and the Ommaya reservoir. The Student's *t*‐test was used for continuous variables and the χ^2^ or Fisher's exact test for categorical variables. All tests of significance were two‐sided; *P*‐values <0.05 were considered statistically significant. Independent variables were selected on the basis of their statistical significance in the univariate analysis, and the criterion for inclusion of a variable in the multivariate analysis was significance level <0.1. All analyses were performed using IBM SPSS software, version 20.0 (IBM Corp., Armonk, NY, USA).

### Patient characteristics

Among the 1189 patients with lung adenocarcinoma harboring EGFR mutations during the study period, 117 with cytologically confirmed LMC were identified. The median age (interquartile range, IQR) was 56.0 (48.5–63.0) years. The study population included 37.1% male and 62.9% female patients. Among all patients, 46.1% presented with distant metastasis at the initial diagnosis. The most common metastatic site was the brain (34.5%), and simultaneous brain parenchymal metastasis was identified in 75.2% of patients with LMC. The clinicopathologic characteristics of the patients are shown in Table 1.

**Table 1:** Baseline characteristics of 1189 patients with lung adenocarcinoma treated with epidermal growth factor receptor‐tyrosine kinase inhibitors (EGFR‐TKIs)

Characteristics	Total (*n* = 1189)	LMC (*n* = 117)	No LMC (*n* = 1072)	*P*‐value
Age (years)	60.0 (53.0–69.0)	56.0 (48.5–63.0)	61.0 (53.0–69.0)	<0.001
Sex				0.746
Male	441 (37.1%)	45 (38.5%)	396 (36.9%)	
Female	748 (62.9%)	72 (61.5%)	676 (63.1%)	
Disease status				<0.001
Initially metastatic	500 (46.1%)	78 (71.6%)	422 (43.2%)	
Recurrent	405 (49.8%)	31 (28.4%)	554 (56.8%)	
Site of metastasis				
Pleura	257 (22.1%)	29 (24.8%)	228 (21.8%)	0.872
Bone	316 (27.2%)	58 (49.6%)	258 (24.7%)	<0.001
Brain	410 (34.5%)	88 (75.2%)	322 (30.0%)	<0.001
Lung	114 (9.8%)	26 (22.2%)	88 (8.4%)	<0.001
Liver	58 (5.0%)	14 (12.0%)	44 (4.2%)	<0.001
Adrenal	33 (2.8%)	7 (6.0%)	26 (2.5%)	0.031
EGFR mutation (exon)				0.218
19	560 (52.2%)	64 (54.7%)	560 (52.2%)	
21	459 (38.6%)	46 (39.3%)	413 (38.5%)	
18	27 (2.3%)	4 (3.4%)	23 (2.1%)	
20	11 (0.9%)	2 (1.7%)	9 (0.8%)	
Double mutation				
19, 21	3 (0.3%)	1 (0.9%)	2 (0.2%)	
18, 21	2 (0.2%)	0 (0.0%)	2 (0.2%)	
19, 20	6 (0.5%)	0 (0.0%)	6 (0.6%)	
20, 21	2 (0.2%)	0 (0.0%)	2 (0.2%)	

Data are reported as n (%) or median (interquartile range [IQR]).

EGFR, epidermal growth factor receptor; LMC, leptomeningeal carcinomatosis.

### Results

The median time from the date of initial diagnosis with lung cancer to the occurrence of LMC was 13.5 months (IQR 6.8–23.6 months). In univariate analysis, younger age, initially metastatic disease and metastasis to the brain, bone, liver, lung, or adrenal glands were associated with LMC occurrence. However, younger age, initially metastatic disease, and metastasis to the brain or lung remained as risk factors related to LMC in multivariate analysis: hazard ratio (HR) 0.958 (95% confidence interval [CI] 0.938–0.979), *P* < 0.001; HR 3.768 (95% CI 2.272–6.249), *P* < 0.001; HR 8.682 (95% CI 5.209–14.472), *P* < 0.001; HR 2.317 (95% CI 1.311–4.096), *P* = 0.004, respectively (Table 2).

**Table 2:** Risk factors for leptomeningeal carcinomatosis (LMC)

	Univariate analysis				Multivariate analysis	
Risk factors	Total *(n* = 1189)	LMC (*n* = 117)	No LMC (*n* = 1072)	*P*‐value	HR (95% CI)	*P*‑value
Age (years)	60.6 ± 10.8	56.1 ± 10.3	61.1 ± 10.8	<0.001	0.958 (0.938–0.979)	<0.001
Initially metastatic disease	500 (46.1%)	78 (71.6%)	422 (43.2%)	<0.001	3.768 (2.272–6.249)	<0.001
Bone metastasis	316 (27.2%)	58 (49.6%)	258 (24.7%)	<0.001	1.232 (0.757–2.005)	0.402
Brain metastasis	410 (34.5%)	88 (75.2%)	322 (30.0%)	<0.001	8.682 (5.209–14.472)	<0.001
Lung metastasis	114 (9.8%)	26 (22.2%)	88 (8.4%)	<0.001	2.317 (1.311–4.096)	0.004
Liver metastasis	58 (5.0%)	14 (12.0%)	44 (4.2%)	<0.001	1.578 (0.719–3.463)	0.256
Adrenal metastasis	33 (2.8%)	7 (6.0%)	26 (2.5%)	0.031	1.162 (0.411–3.287)	0.777

Data are reported as *n* (%) or mean ± standard deviation.

CI, confidence interval; HR, hazard ratio; LMC, leptomeningeal carcinomatosis.

### Survival outcomes according to treatment modality

**Figure 1:**
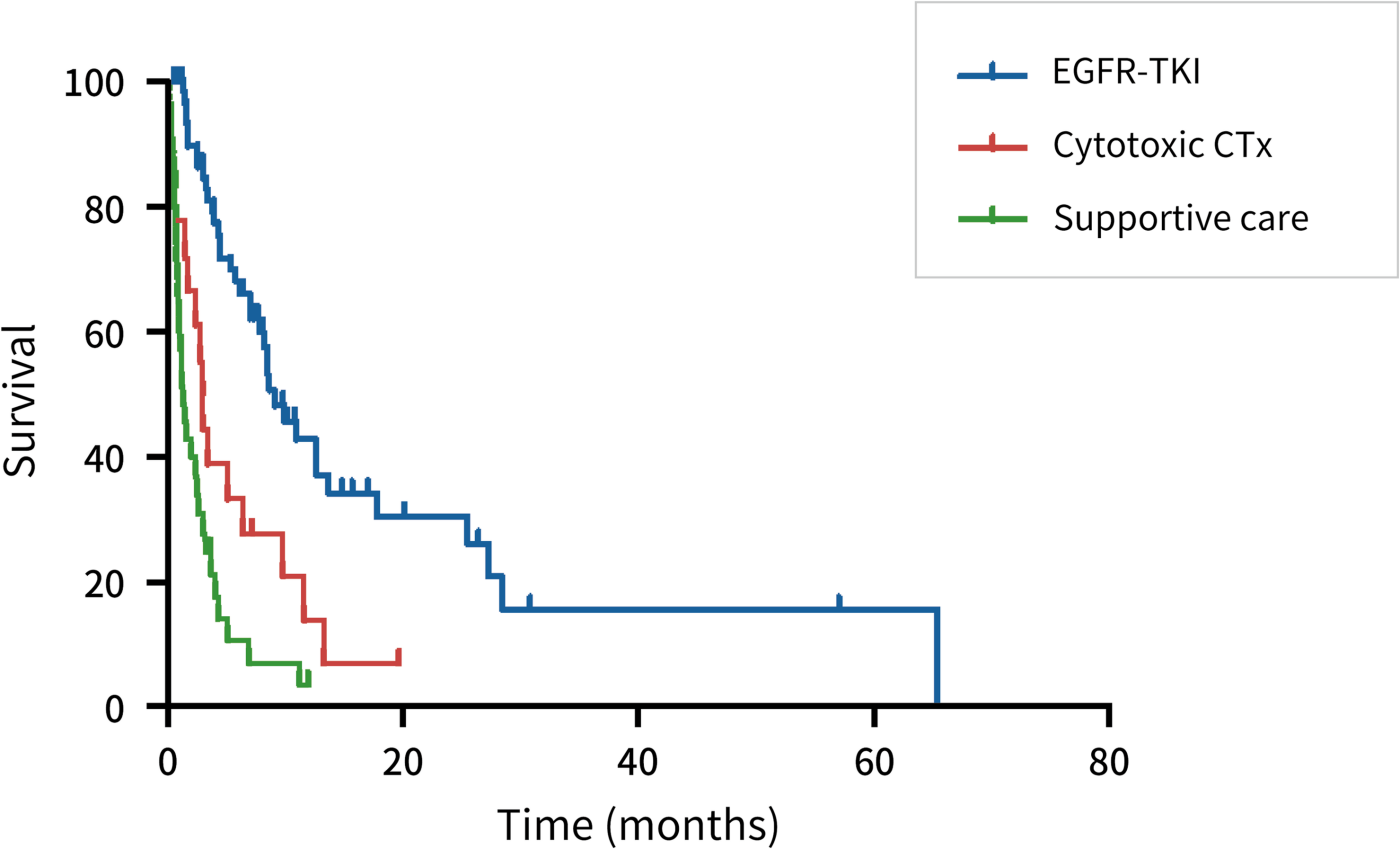
Kaplan‐Meier curve according to treatment modality: EGFR‐TKIs versus cytotoxic chemotherapy versus supportive care. EGFR‐TKIs, epidermal growth factor receptor tyrosine kinase inhibitors.

**Figure 2:**
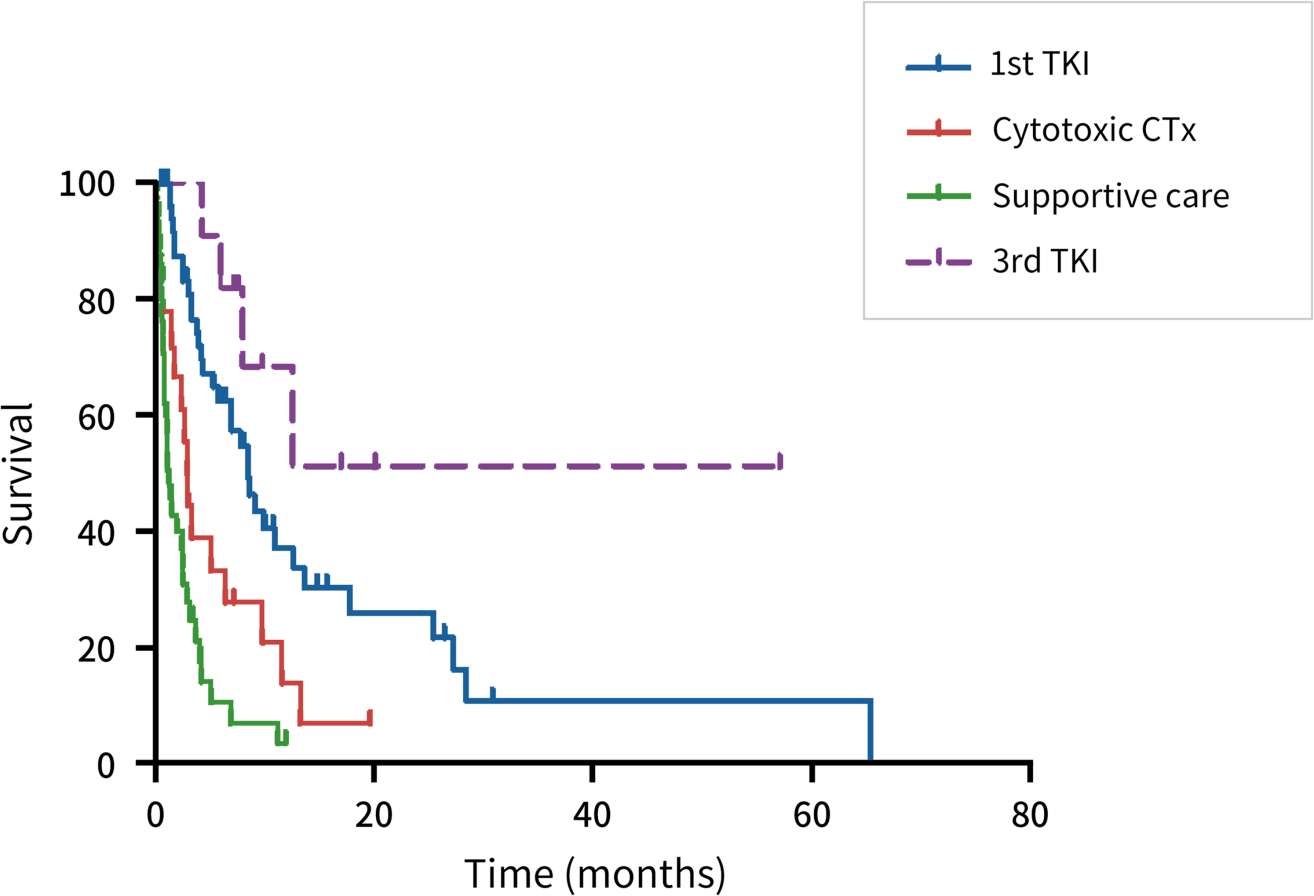
Kaplan‐Meier curve according to treatment modality. First‐generation EGFR‐TKIs versus third‐generation EGFR‐TKIs versus cytotoxic chemotherapy versus supportive care. EGFR‐TKIs, epidermal growth factor receptor tyrosine kinase inhibitors.

The median survival time from the date of LMC documentation was 3.8 months (IQR 1.5–8.6 months). The baseline characteristics of the three groups, according to treatment mortality, are shown in Table 3. In the subgroup analysis of these groups, the median survival time was significantly longer in patients treated with EGFR‐TKIs (7.1 months, IQR 3.3–11.4 months), followed by those treated with cytotoxic chemotherapy (3.1 months, IQR 1.3–7.9 months) and with best supportive care (1.2 months, IQR 0.7–3.2 months) (Fig 1). The survival of patients treated with third‐generation EGFR‐TKIs was significantly longer than the other treatment groups (Fig 2). In multivariate analysis, Eastern Cooperative Oncology Group (ECOG) performance status score of two or less, treatment with EGFR‐TKIs, and insertion of an Ommaya reservoir were significantly associated with favorable outcomes in terms of OS (Table 4).

**Table 3:** Baseline characteristics of 117 patients with leptomeningeal carcinomatosis (LMC) from lung adenocarcinoma according to treatment modality

Characteristics	Total (*n* = 117)	EGFR‐TKI (*n* = 62)	Cytotoxic CTx (*n* = 19)	Supportive care (*n* = 36)	*P‐*value
Age (years)	56.0 (48.5–63.0)	56.0 (50.0–62.0)	56.0 (44.0–61.0)	57.0 (47.3–66.0)	0.611
Sex					0.474
Male	45 (38.5%)	25 (40.3%)	5 (26.3%)	15 (41.7%)	
Female	72 (61.5%)	37 (59.7%)	14 (73.7%)	21 (58.3%)	
Smoking history					0.614
Current or ex‐smoker	42 (35.9%)	22 (35.5%)	5 (26.3%)	15 (41.7%)	
Never	75 (64.1%)	40 (64.5%)	14 (73.7%)	21 (58.3%)	
Disease status					0.003
Initially metastatic	78 (71.6%)	37 (60.7%)	13 (76.5%)	28 (90.3%)	
Recurrent	31 (28.4%)	24 (39.3%)	4 (23.5%)	3 (9.7%)	
Brain metastasis with LMS	88 (75.9%)	50 (82.0%)	14 (73.7%)	24 (66.7%)	0.087
Systemic disease status					0.001
Stable	54 (79.4%)	48 (88.9%)	3 (37.5%)	3 (50.0%)	
Progressive	14 (20.6%)	6 (11.1%)	5 (62.5%)	3 (50.0%)	
ECOG PS					0.072
≤2	81 (69.2%)	48 (77.4%)	11 (57.9%)	22 (61.1%)	
>2	36 (30.8%)	14 (22.6%)	8 (42.1%)	14 (38.9%)	
Time interval to LMC (months)	13.5 (6.8–23.6)	9.4 (0.4–23.8)	14.8 (8.6–21.2)	15.0 (9.6–26.6)	0.486
Presenting symptoms					
Headache	60 (51.7%)	35 (57.4%)	11 (57.9%)	14 (38.9%)	
Cauda equina	14 (12.1%)	4 (6.6%)	5 (26.3%)	5 (13.9%)	
Dizziness	16 (13.8%)	10 (16.4%)	0 (0.0%)	6 (16.7%)	
Altered mentality	15 (12.9%)	6 (9.8%)	1 (5.3%)	8 (22.2%)	
Seizure	7 (6.0%)	5 (8.2%)	2 (10.5%)	0 (0.0%)	
Memory impairment	2 (1.7%)	1 (1.6%)	0 (0.0%)	1 (2.8%)	
Combined RTx					
Whole brain	25 (21.4%)	15 (24.2%)	3 (15.8%)	7 (19.4%)	0.536
GKRS	23 (19.7%)	11 (17.7%)	3 (15.8%)	9 (25.0%)	0.417
Ommaya reservoir	67 (57.3%)	41 (66.1%)	9 (47.4%)	17 (47.2%)	0.056
IT chemotherapy	35 (29.9%)	23 (37.1%)	2 (10.5%)	10 (27.8%)	0.237

Data are reported as *n* (%) or median (interquartile range [IQR]).

CTx, chemotherapy; ECOG PS, Eastern Cooperative Oncology Group performance status; EGFR, epidermal growth factor receptor; GKRS, Gamma Knife radiosurgery; IT, intrathecal; LMC, leptomeningeal carcinomatosis; RTx, radiotherapy; TKI, tyrosine kinase inhibitor.

**Table 4:** Risk factors for death after diagnosis of leptomeningeal carcinomatosis (LMC)

	Univariate analysis			Multivariate analysis		
Characteristics	HR	95% CI	*P*‐value	HR	95% CI	*P*‐value
Sex (F/M)	0.826	0.533–1.281	0.393	—		
Age	1.014	0.991–1.037	0.238	—		
Smoking	1.146	0.730–1.798	0.553	—		
Recurrent disease	0.586	0.345–0.996	0.048	0.719	0.400–1.291	0.269
ITC	0.692	0.422–1.134	0.144	1.144	0.615–2.128	0.671
ICP controlled	0.919	0.467–1.809	0.807	—		
CSF negative conversion	0.664	0.367–1.201	0.176	—		
ECOG PS >2	1.669	1.065–2.616	0.026	1.979	1.200–3.263	0.007
Treatment modalities						
EGFR‐TKI	1	—	<0.001	1		<0.001
Cytotoxic chemotherapy	2.553	1.401–4.653	0.002	2.222	1.146–4.308	0.018
Best supportive care	5.471	3.269–9.157	<0.001	5.638	3.153–10.080	<0.001
Ommaya reservoir	0.531	0.346–0.815	0.004	0.445	0.255–0.779	0.005

CI, confidence interval; CSF, cerebrospinal fluid; EGFR, epidermal growth factor receptor; ECOG PS, Eastern Cooperative Oncology Group performance status; F/M, female/male; HR, hazard ratio; ICP, intracranial pressure; ITC, intrathecal chemotherapy; TKI, tyrosine kinase inhibitor.

### Neurologic outcomes of IT chemotherapy and Ommaya reservoir

The median number of cycles of IT chemotherapy was eight (IQR 5.0–11.0). The most common initial presenting symptom was headache (51.7%), followed by dizziness (13.8%), altered mentality (12.9%), and cauda equina (12.1%). IT chemotherapy was related to improvement of neurologic symptoms as well as CSF negative conversion (*P* = 0.013, 0.004, respectively; see Appendix Table A1); these findings were also observed in the analysis of patients with an Ommaya reservoir (*P* = 0.002, 0.013, respectively; see Appendix Table A1).

## Discussion

To the best of our knowledge, this is the first study to investigate the risk factors and predictive outcomes of patients with stage 4 lung adenocarcinoma harboring EGFR mutations. The most important finding was that EGFR‐TKI treatment showed good efficacy that was superior to cytotoxic chemotherapy. In particular, third‐generation EGFR‐TKIs conferred survival benefits as compared with other treatments. The second important finding was that IT chemotherapy could relieve neurologic symptoms and was associated with CSF negative conversion, although it did not affect survival in patients with LMC. Of note, an Ommaya reservoir was an independent positive prognostic factor and had a similar effect on neurologic outcomes as that of IT chemotherapy. The third notable finding in this study was that brain parenchymal metastasis was revealed as the most significant factor related to LMC.

Pharmacokinetic and pharmacodynamic data have shown that only 2%–13% of concentrations are detected in CSF compared with plasma when first‐ or second‐generation EGFR‐TKIs are administered in a standard dose.^15^ Several studies have reported higher concentrations in the CSF of erlotinib than those of gefitinib, regardless of the dose.^15^, ^16^ In addition, third‐generation EGFR‐TKIs, which can reach therapeutic concentrations in the CSF, are associated with a survival benefit.^17^ In the current study, the effect of EGFR‐TKIs on prolongation of survival was significantly better than that of cytotoxic chemotherapy or best supportive care, despite administration of a standard dose of EGFR‐TKIs. Moreover, in subgroup analysis of EGFR‐TKIs, there was no significant difference in OS between erlotinib and gefitinib (8.7 months [95% CI 5.0–12.3 months] vs. 8.5 months [95% CI 5.1–11.9 months], *P* = 0.608; data not shown). This survival benefit was observed in patients with ECOG PS ≤2 and controlled extracranial disease, consistent with previous studies.^18^, ^19^, ^20^ Moreover, of 62 patients treated with EGFR‐TKIs, 11 (17.7%) received third‐generation EGFR‐TKIs, and the survival outcomes in these patients were significantly longer than with other treatments, including first‐generation EGFR‐TKIs and cytotoxic chemotherapy. These findings support the efficacy of third‐generation EGFR‐TKIs in patients with LMC, which is in line with previously published data.^21^, ^22^ Approval of third‐generation EGFR‐TKIs as a first‐line chemotherapeutic regimen in EGFR‐mutant adenocarcinoma will confer a further survival benefit in patients with LMC from NSCLC.

There are limited data on risk factors for LMC. In the current study, data of approximately 1200 patients were analyzed to determine factors associated with LMC. In our study, brain metastasis was the most important predictive factor of LMC. Approximately 35% of patients with LMC were simultaneously diagnosed with brain metastasis, which is a smaller proportion than that in a study by Li and colleagues.^23^ A possible explanation for this discrepancy is that LMC was diagnosed using a CSF cytologic test in our study, but the previous study defined LMC with confirmation using a cytologic test or magnetic resonance imaging (MRI). A notable finding is that metastasis to a different lobe of the lung was also revealed as an independent factor for LMC. Apart from the above findings, younger age and presenting with initially distant metastatic disease rather than recurrent lung cancer were related to the development of LMC. Given that false‐negative results occur in 20%–30% of MRI studies,^10^ our finding suggests that patients who have these risk factors might benefit from CSF analysis.

In a pooled analysis performed by Wu *et al*. IT chemotherapy was linked to longer survival duration than with multiple interventions.^12^ However, the optimal protocol of IT chemotherapy has not yet been established.^9^ Other feasible options in treating LMC include whole brain radiotherapy (WBRT) and gamma knife radiosurgery (GKRS). In the current study, IT chemotherapy was regarded as adjunctive treatment, along with WBRT or GKRS. Although there were no consistent effects of IT chemotherapy, WBRT, or GKRS,^24^, ^25^ survival benefits of these adjunctive therapies were not observed in our analysis. However, interestingly, the Ommaya reservoir showed a prognostic effect on survival. Considering the study by Gwak and colleagues,^26^ who stated that uncontrolled ICP was related to an unfavorable outcome in patients with NSCLC, controlling ICP with an Ommaya reservoir might affect patient survival. However, there was limited available information on the opening CSF pressure due to the retrospective nature of the current study.

There were several limitations in our study. First, this study was conducted in a single center using a retrospective design. LMC is a rare complication of lung cancer and has low prevalence; however, our study included a relatively large number of patients, thereby providing firm clinical evidence of treatment for LMC. Second, we arbitrarily set the cutoff value for IT chemotherapy as one month or more, but there was a lack of related evidence. Third, although most patients were examined in a follow‐up CSF study, only eight patients who underwent subsequent CSF examination met the Response Assessment in Neuro‐Oncology criteria, which is maintenance of cytologic conversion for four weeks.^27^ Fourth, according to a report by Shigeki *et al*., detection of the EGFR T790M mutation in the CSF is related to the response to third‐generation EGFR‐TKIs^22^; however, molecular analysis of CSF was not performed in our study.

In conclusion, younger age, initially metastatic disease, and metastasis to the brain or another lobe of the lung were associated with LMC in patients with lung adenocarcinoma harboring EGFR mutation. Treating these patients with EGFR‐TKI or cytotoxic chemotherapy, insertion of an Ommaya reservoir, and good performance status showed favorable survival outcomes. Moreover, IT chemotherapy and the Ommaya reservoir were significantly associated with improved neurologic outcome, including symptoms and signs, and with CSF negative conversion.

## Disclosure

No authors report any conflict of interest.

## Supporting information


**Appendix S1**: Supporting information

## References

[1] Bray F , Ferlay J , Soerjomataram I , Siegel RL , Torre LA , Jemal A . Global cancer statistics 2018: GLOBOCAN estimates of incidence and mortality worldwide for 36 cancers in 185 countries. CA Cancer J Clin 2018; 68: 394–424. 30207593 10.3322/caac.21492

[2] Siegel RL , Miller KD , Jemal A . Cancer statistics, 2017. CA Cancer J Clin 2017; 67: 7–30. 28055103 10.3322/caac.21387

[3] Mok TS , Wu YL , Thongprasert S *et al* Gefitinib or carboplatin‐paclitaxel in pulmonary adenocarcinoma. N Engl J Med 2009; 361: 947–57. 19692680 10.1056/NEJMoa0810699

[4] Maemondo M , Inoue A , Kobayashi K *et al* Gefitinib or chemotherapy for non‐small‐cell lung cancer with mutated EGFR. N Engl J Med 2010; 362: 2380–8. 20573926 10.1056/NEJMoa0909530

[5] Rosell R , Carcereny E , Gervais R *et al* Erlotinib versus standard chemotherapy as first‐line treatment for European patients with advanced EGFR mutation‐positive non‐small‐cell lung cancer (EURTAC): A multicentre, open‐label, randomised phase 3 trial. Lancet Oncol 2012; 13: 239–46. 22285168 10.1016/S1470-2045(11)70393-X

[6] Park CK , Kim SJ . Trends and updated statistics of lung cancer in Korea. Tuberc Respir Dis (Seoul) 2019; 82: 175–7. 30915782 10.4046/trd.2019.0015 PMC6435935

[7] Park JY , Jang SH . Epidemiology of lung cancer in Korea: Recent trends. Tuberc Respir Dis (Seoul) 2016; 79: 58–69. 27064578 10.4046/trd.2016.79.2.58 PMC4823185

[8] Kim YC , Won YJ . The development of the Korean lung cancer registry (KALC‐R). Tuberc Respir Dis (Seoul) 2019; 82: 91–3. 30302952 10.4046/trd.2018.0032 PMC6435934

[9] Cheng H , Perez‐Soler R . Leptomeningeal metastases in non‐small‐cell lung cancer. Lancet Oncol 2018; 19: e43–55. 29304362 10.1016/S1470-2045(17)30689-7

[10] Remon J , Le Rhun E , Besse B . Leptomeningeal carcinomatosis in non‐small cell lung cancer patients: A continuing challenge in the personalized treatment era. Cancer Treat Rev 2017; 53: 128–37. 28110254 10.1016/j.ctrv.2016.12.006

[11] Iuchi T , Shingyoji M , Itakura M *et al* Frequency of brain metastases in non‐small‐cell lung cancer, and their association with epidermal growth factor receptor mutations. Int J Clin Oncol 2015; 20: 674–9. 25336382 10.1007/s10147-014-0760-9

[12] Wu YL , Zhou L , Lu Y . Intrathecal chemotherapy as a treatment for leptomeningeal metastasis of non‐small cell lung cancer: A pooled analysis. Oncol Lett 2016; 12: 1301–14. 27446430 10.3892/ol.2016.4783 PMC4950629

[13] Shin SY , Park YR , Shin Y *et al* A De‐identification method for bilingual clinical texts of various note types. J Korean Med Sci 2015; 30: 7–15. 25552878 10.3346/jkms.2015.30.1.7 PMC4278030

[14] Shin SY , Lyu Y , Shin Y *et al* Lessons learned from development of de‐identification system for biomedical research in a Korean Tertiary Hospital. Healthc Inform Res 2013; 19: 102–9. 23882415 10.4258/hir.2013.19.2.102 PMC3717433

[15] Togashi Y , Masago K , Masuda S *et al* Cerebrospinal fluid concentration of gefitinib and erlotinib in patients with non‐small cell lung cancer. Cancer Chemother Pharmacol 2012; 70: 399–405. 22806307 10.1007/s00280-012-1929-4

[16] Clarke JL , Pao W , Wu N , Miller VA , Lassman AB . High dose weekly erlotinib achieves therapeutic concentrations in CSF and is effective in leptomeningeal metastases from epidermal growth factor receptor mutant lung cancer. J Neurooncol 2010; 99: 283–6. 20146086 10.1007/s11060-010-0128-6 PMC3973736

[17] Nanjo S , Hata A , Okuda C *et al* Standard‐dose osimertinib for refractory leptomeningeal metastases in T790M‐positive EGFR‐mutant non‐small cell lung cancer. Br J Cancer 2017; 118: 32. 29190637 10.1038/bjc.2017.394 PMC5765232

[18] Park JH , Kim YJ , Lee J‐O *et al* Clinical outcomes of leptomeningeal metastasis in patients with non‐small cell lung cancer in the modern chemotherapy era. Lung Cancer 2012; 76: 387–92. 22186628 10.1016/j.lungcan.2011.11.022

[19] Kim H , Lee EM . A retrospective analysis of the clinical outcomes of leptomeningeal metastasis in patients with solid tumors. Brain Tumor Res Treat 2018; 6: 54–9. 30381917 10.14791/btrt.2018.6.e12 PMC6212684

[20] Lee SJ , Lee J‐I , Nam D‐H *et al* Leptomeningeal carcinomatosis in non‐small‐cell lung cancer patients: Impact on survival and correlated prognostic factors. J Thorac Oncol 2013; 8: 185–91. 23328548 10.1097/JTO.0b013e3182773f21

[21] Saboundji K , Auliac JB , Perol M *et al* Efficacy of osimertinib in *EGFR*‐mutated non‐small cell lung cancer with leptomeningeal metastases pretreated with EGFR‐tyrosine kinase inhibitors. Target Oncol 2018; 13: 501–7. 30039345 10.1007/s11523-018-0581-2

[22] Nanjo S , Hata A , Okuda C *et al* Standard‐dose osimertinib for refractory leptomeningeal metastases in T790M‐positive EGFR‐mutant non‐small cell lung cancer. Br J Cancer 2018; 118: 32–7. 29190637 10.1038/bjc.2017.394 PMC5765232

[23] Li YS , Jiang BY , Yang JJ *et al* Leptomeningeal metastases in patients with NSCLC with EGFR mutations. J Thorac Oncol 2016; 11: 1962–9. 27539328 10.1016/j.jtho.2016.06.029

[24] Liao BC , Lee JH , Lin CC *et al* Epidermal growth factor receptor tyrosine kinase inhibitors for non‐small‐cell lung cancer patients with leptomeningeal carcinomatosis. J Thorac Oncol 2015; 10: 1754–61. 26334749 10.1097/JTO.0000000000000669

[25] Morris PG , Reiner AS , Szenberg OR *et al* Leptomeningeal metastasis from non‐small cell lung cancer: Survival and the impact of whole brain radiotherapy. J Thorac Oncol 2012; 7: 382–5. 22089116 10.1097/JTO.0b013e3182398e4f

[26] Gwak HS , Joo J , Kim S *et al* Analysis of treatment outcomes of intraventricular chemotherapy in 105 patients for leptomeningeal carcinomatosis from non‐small‐cell lung cancer. J Thorac Oncol 2013; 8: 599–605. 23422833 10.1097/JTO.0b013e318287c943

[27] Chamberlain M , Junck L , Brandsma D *et al* Leptomeningeal metastases: A RANO proposal for response criteria. Neuro Oncol 2017; 19: 484–92. 28039364 10.1093/neuonc/now183 PMC5464328

