# Supplementary material for: Impact of clinicopathologic features on leptomeningeal metastasis from lung adenocarcinoma and treatment efficacy with epidermal growth factor receptor tyrosine kinase inhibitor
Source: Thorac Cancer. 2020 Jan 7;11(2):436–42. doi: 10.1111/1759-7714.13296 (PMC6996974; doi:10.1111/1759-7714.13296)
Supplement: Supplementary file 1 — Appendix S1: Supporting information [file TCA-11-436-s001.docx]

**Supplementary material**

Impact of clinicopathologic features on leptomeningeal metastasis from lung adenocarcinoma and treatment efficacy with epidermal growth factor receptor tyrosine kinase inhibitor

**Authors**

Byoung Soo Kwon^[[1]](#footnote-1),a^, Young Hyun Cho^2^, Shin-Kyo Yoon^3^, Dae Ho Lee^3^, Sang-We Kim^3^, Do Hoon Kwon^2^, Jae Cheol Lee^3^, Chang-Min Choi^1,3^

^1^Department of Pulmonology and Critical Care Medicine, University of Ulsan College of Medicine, Asan Medical Center, Seoul, South Korea

^2^Department of Neurosurgery, University of Ulsan College of Medicine, Asan Medical Center, Seoul, South Korea

^3^Department of Oncology, University of Ulsan College of Medicine, Asan Medical Center, Seoul, South Korea

**Table of content**

Table A. 1 Page 3

**Table A. 1.** Efficacy of intrathecal chemotherapy and Ommaya reservoir in patients with leptomeningeal seeding from lung adenocarcinoma

|  |  | Total (n=115) | Relieved | Not relieved | *P* value |
| --- | --- | --- | --- | --- | --- |
| Symptoms relieved | ITC | 35 (30.4%) | 17 (45.9%) | 18 (23.1%) | 0.013 |
|  | Ommaya | 66 (57.4%) | 29 (78.4%) | 37 (47.4%) | 0.002 |
|  |  | Total (n=83) | Converted | Not converted | *P* value |
| CSF negative conversion | ITC | 35 (42.2%) | 17 (65.4%) | 18 (31.6%) | 0.004 |
|  | Ommaya | 62 (74.7%) | 24 (92.3%) | 38 (66.7%) | 0.013 |

ITC, intrathecal chemotherapy; CSF, cerebrospinal fluid.

Data are reported as n (%).

1. aDivision of Pulmonary and Critical Care Medicine, Department of Internal Medicine, Seoul National University Bundang Hospital, Seongnam, Gyeonggi-Do 13620, South Korea [↑](#footnote-ref-1)
